# Comparative Analysis of BIOCHIP Mosaic-Based Indirect Immunofluorescence with Enzyme-Linked Immunosorbent Assay for Diagnosing Myasthenia Gravis

**DOI:** 10.3390/diagnostics11112098

**Published:** 2021-11-13

**Authors:** Caterina Maria Gambino, Luisa Agnello, Bruna Lo Sasso, Concetta Scazzone, Rosaria Vincenza Giglio, Giuseppina Candore, Anna Maria Ciaccio, Vincenzo Di Stefano, Filippo Brighina, Matteo Vidali, Marcello Ciaccio

**Affiliations:** 1 Clinical Molecular Medicine and Laboratory Medicine, Institute of Clinical Biochemistry, Department of Biomedicine, Neurosciences and Advanced Diagnostics, University of Palermo, 90127 Palermo, Italy; cmgambino@libero.it (C.M.G.); luisa.agnello@unipa.it (L.A.); bruna.losasso@unipa.it (B.L.S.); concetta.scazzone@unipa.it (C.S.); giglio.rosaria.vincenza@gmail.com (R.V.G.); giuseppina.candore@unipa.it (G.C.); 2 Unit of Clinical Biochemistry, University of Palermo, 90127 Palermo, Italy; annamaria.ciaccio@unipa.it; 3 Unit of Neurology, Department of Biomedicine, Neurosciences and Advanced Diagnostics, 90127 Palermo, Italy; vincenzo.distefano07@unipa.it (V.D.S.); filippo.brighina@unipa.it (F.B.); 4 Foundation IRCCS Ca’ Granda Ospedale Maggiore Policlinico, 20122 Milan, Italy; matteo.vidali@gmail.com; 5 Department of Laboratory Medicine, Azienda Ospedaliera Universitaria Policlinico “P. Giaccone”, 90127 Palermo, Italy

**Keywords:** myasthenia gravis, diagnosis, biomarker, anti-acetylcholine receptor antibodies, anti-muscle-specific tyrosine kinase antibodies, BIOCHIP

## Abstract

Background: The detection of anti-acetylcholine receptor (AChR) and anti-muscle-specific tyrosine kinase (MuSK) antibodies is useful in myasthenia gravis (MG) diagnosis and management. BIOCHIP mosaic-based indirect immunofluorescence is a novel analytical method, which employs the simultaneous detection of anti-AChR and anti-MuSK antibodies in a single miniature incubation field. In this study, we compare, for the first time, the BIOCHIP MG mosaic with conventional enzyme-linked immunosorbent assay (ELISA) in the diagnosis of MG. Methods: A total of 71 patients with MG diagnosis were included in the study. Anti-AChR and anti-MuSK antibodies were measured separately by two different ELISA and simultaneously by BIOCHIP. The results were then compared. Results: The overall concordance between ELISA and BIOCHIP for anti-AChR reactivity was 74%. Cohen’s kappa was 0.51 (95% CI 0.32–0.71), which corresponds to 90% of the maximum possible kappa (0.57), given the observed marginal frequencies. The overall concordance for anti-MuSK reactivity was 84%. Cohen’s kappa was 0.11 (95% CI 0.00–0.36), which corresponds to 41% of the maximum possible kappa (0.27). Conclusion: The overall concordance among assays is not optimal.

## 1. Introduction

Myasthenia gravis (MG) is a chronic autoimmune disease characterized by antibody-mediated reduction of neuromuscular transmission resulting in skeletal muscle weakness and fatigue [1,2,3]. The disease onset is characterized by the weakness of eye muscles, which leads to diplopia and/or ptosis. About 20% of patients present only eye muscle symptoms, commonly referred to as ocular MG (OMG), while most patients develop other skeletal muscles symptoms within a few years, leading to generalized MG (GMG). The disease onset is characterized by 2 peaks of incidence: early-onset and late-onset, which commonly occurs in women of up to 40 years of age and in men of around 50 years of age, respectively [4].

The detection of autoantibodies is crucial for MG diagnosis, prognosis, and monitor response to treatment [5].

Autoantibodies against the nicotinic acetylcholine receptor (AChR) localized on the post-synaptic membrane of the neuromuscular junction are highly specific to MG. They are pathogenic and are detectable in approximately 85% of MG patients [6]. In approximately 6% of MG patients, the autoimmune response is against the muscle-specific tyrosine kinase (MuSK), a protein belonging to the clustering of AChR and other postsynaptic components of the neuromuscular junction [7]. Anti-MuSK autoantibodies are also pathogenic. They are mainly detectable in younger women and are associated with a severe disease course [8]. Recently, the low-density lipoprotein receptor-related protein 4 (LRP4), a molecule that forms a complex with MuSK, has been described as a novel auto-antigenic target in about 2% of seronegative MG patients [6], but its clinical significance is still unknown.

Since their discovery, several assays have been developed and commercialized for the detection of AChR and MuSK autoantibodies, including the radioimmunoprecipitation assay (RIPA) and enzyme-linked immunosorbent assay (ELISA) [9,10,11,12,13]. Although such methods are characterized by their high diagnostic accuracy, they are laborious and, especially for RIPA, the use of isotopes containing radioactivity has discouraged many laboratories. Moreover, ELISA methods have the disadvantage of being impractical when only a few samples must be analyzed. Different ELISA assays are commercially available and are based either on direct or on competitive methods, leading to discordant results among laboratories. Indeed, incongruous results have often been reported for identical samples tested with different assays. In addition, there is always the need to run two different antibody assays (e.g., anti-AChR and MuSK) to test a complete antibody panel. Finally, the sensitivity of antibody detection is limited because some antibodies may bind more effectively to the antigen when expressed on the cell surface rather than recombinant or soluble antigens.

The present study aims to compare a new commercially available multiparametric indirect immunofluorescence (IIF) assay, noted as the BIOCHIP mosaic, with conventional ELISA for the detection of anti-AChR and anti-MuSK autoantibodies for the diagnosis of MG. The BIOCHIP mosaic employs the simultaneous detection of anti-AChR and anti-MuSK autoantibodies in a single miniature incubation field. Thus, it is fast to perform and highly reproducible, with great potential in clinical practice by avoiding the use of radioactive materials and reducing the turn-around time (TAT).

## 2. Materials and Methods

### 2.1. Study Population

We performed an observational retrospective study on consecutive patients with MG enrolled at the Unit of Neurology at the University Hospital Policlinico “P. Giaccone” of Palermo, Italy.

The demographic and clinical data of the patients, including sex, age at onset, initial symptoms, presence of MG crisis, the severity of MG based on the Myasthenia Gravis Foundation of America (MGFA) clinical classification, and the state of immunosuppressive treatment, were recorded by reviewing medical records.

The diagnosis of MG was made according to the International Consensus Guidance for Management of MG [14], including the following criteria: a diffuse weakness with or without ocular and respiratory involvement; decremented U-shaped response at 3 Hz repetitive nerve stimulation and/or increased jitter at single-fiber electromyography; and exclusion of any other neurologic or inflammatory condition. All patients were screened for the presence of thymoma with computed tomography (CT) or/and magnetic resonance (MR) imaging scanning of the mediastinum.

The study was conducted in accordance with the ethical standards as formulated in the Helsinki Declaration. All patients remained anonymous and gave informed consent to be included in the study.

### 2.2. Biochemical Analysis

The blood samples of the patients were collected in dry tubes and the serum obtained after centrifugation was stored at −80 °C until analysis.

Anti AChR and MuSK autoantibodies were measured by two different assays: a new commercially available BIOCHIP method (MG mosaics, Euroimmun, Lübeck, Germany), which measures antibodies against AChR and MuSK simultaneously, and two different commercially available ELISA methods for detecting AChR (RSR Ltd., Cardiff, UK) and MuSK autoantibodies (IBL International GmbH, Männedorf, Germany).

All analyses were performed at the Institute of Clinical Biochemistry, Clinical Molecular Medicine and Clinical Laboratory Medicine at the University of Palermo.

#### 2.2.1. BIOCHIP Mosaic Indirect Immunofluorescence Assay

The BIOCHIP mosaic-based IIF method for detecting autoantibodies against AChR and MuSK relies on the combination of different substrates put on each field that enables the simultaneous detection of different antibodies. In detail, each field of the slide is a mosaic of 4 substrates: (1) EU90 recombinant cells transfected with the adult acetylcholine receptor (AChR-E); (2) EU90 recombinant cells transfected with fetal acetylcholine receptor (AChR-G); (3) EU90 recombinant cells transfected with MuSK; and (4) EU90 recombinant cells not transfected used as a negative control. This test is designed exclusively for the in vitro determination of human antibodies in serum or plasma.

The assays were performed at a serum dilution of 1:10 in a sample buffer. If the reaction was positive, specific antibodies of classes IgA, IgG, and IgM bound the antigens. In the second and third steps, the attached antibodies were stained with biotin-labeled anti-human antibodies, followed by fluorescein isothiocyanate-labeled avidin and made visible with the fluorescence microscope. Positive and negative control sera provided by the manufacturer were tested in each working session to evaluate run validity, in order to generate a valid result.

Slides were analyzed under a DMIRE2 Leica fluorescence microscope (Leica, Milan, Italy) with a 10× lens. Pictures were acquired with a digital camera model DC250 Leica, using the acquisition software Qfluor550 Leica. Two expert operators, who worked independently and were blinded to the clinical data, interpreted the results. Samples were scored positive if a fluorescence reaction was observed at 1:10 sample dilution.

We also assessed intra-laboratory reproducibility by running positive and negative control samples 10 times in separate runs. Moreover, each serum sample was tested in our laboratory twice by two different operators in a blinded fashion (CMG and GC).

#### 2.2.2. ELISA Methods

The RSR AChR Autoantibody (AChRAb) ELISA kit was used for measuring anti-AChR antibodies. This assay depended on the ability of patient serum AChRAb to compete with 3 different AChR monoclonal antibodies (MAbs 1–3) for binding sites on fetal and adult-type AChR. One MAb (MAb1) was coated onto ELISA plate wells and the other two were labeled with biotin and used in the assay in the liquid phase. In the absence of AChRAb, a sandwich was formed among MAb1, the AChR, and the two biotinylated MAbs. The bind of Mab2- and Mab3-biotin were then detected by the addition of streptavidin peroxidase, which bound specifically to biotin. In the presence of serum AChRAb, the formation of the sandwich was inhibited, and the amount of biotinylated MAbs bound to the plate wells was reduced. A higher concentration of serum AChRAb was associated with a greater inhibition of MAb-biotin binding.

Anti-MuSK antibodies were measured according to the manufacturer’s instructions. Briefly, serum patient antibodies bound to the antigen-coated wells and were detected by a signal amplification system. The substrate reaction was catalyzed via the alkaline phosphate coupled detection antibody. The intensity of the color developed was proportional to the number of patient antibodies detected. All assays were performed in duplicate, with the final concentration calculated by averaging the results. The results were reported as negative (<0.4 nmol/L), borderline (≥0.40 and <0.50 nmol/L), or positive (≥0.50 nmol/L).

### 2.3. Statistical Analysis

Statistical analyses were performed using SPSS statistical software v.17.0 (SPSS Inc., Chicago, IL, USA) and R Language v.4.0.3 (R Foundation for Statistical Computing, Vienna, Austria). The normality distribution was assessed preliminarily by the q–q plot and Shapiro–Wilk test. Quantitative variables were expressed by the median and interquartile range (IQR), while categorical variables were expressed as absolute frequencies and percentages. The concordance was evaluated by concordant pairs and unweighted Cohen’s kappa with 95% CI. Kappa was also reported as the proportion of maximum possible, given the observed marginal frequencies.

## 3. Results

In this study, we included a total of 71 Caucasian patients with confirmed MG, 40 males, ages ranging from 31 to 83 years, and 31 females, ages ranging from 18 to 78 years.

At the time of enrolment, among all MG patients, 10 (14%) had thymoma, 9 (13%) used symptomatic monotherapy with cholinesterase inhibitors, and the majority (55%) additionally used oral corticosteroids and/or steroid-sparing immunosuppressive therapy (29%).

**Figure 1:**
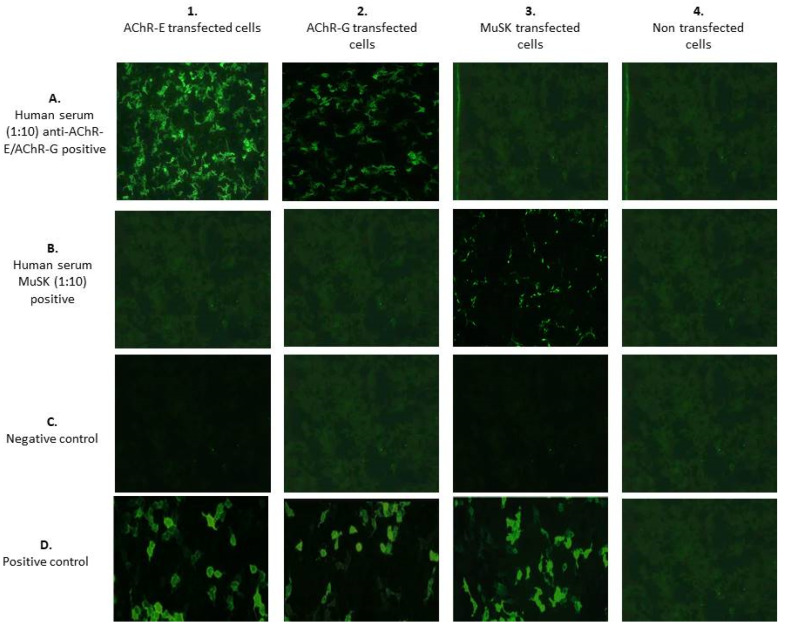
BIOCHIP mosaic for MG diagnosis. On a standard-sized slide, there are five incubation fields, each with four different substrates. (**1**) Anti-adult acetylcholine receptor (AChR-E) positive transfected cells; (**2**) anti-fetal acetylcholine receptor (AChR-G) positive transfected cells; (**3**) anti-MuSK positive transfected cells; and (**4**) no transfected cells.

The BIOCHIP mosaic method provided a qualitative evaluation of circulating antibodies. All samples were tested at 1:10 dilution, in accordance with the manufacturer’s recommendations. Negative samples were defined by the complete absence of staining in all four substrates (Figure 1, row C). However, as the use of only a single dilution may lead to blocking or masking effects in high-titered sera, causing false–negative results, sera that were negative at 1:10 were retested at 1:100 dilutions. At this dilution, no pro-zone effects (defined as the lack of detection of antibodies in low dilutions, but their presence in high dilution) were observed, confirming that those sera were true negatives.

Serum reactivity was evaluated as follows:

- Antibodies against AChR-E showed a flat or fine-to-coarse granular fluorescence of the cells, and also a smooth to very fine granular fluorescence of the cells in the absence of the staining of the membrane of the non-transfected cells. The area of the cell nucleus was only slightly stained (Figure 1(1A)).
- Antibodies against AChR-G showed a spotted, and also a smooth-to-very fine granular fluorescence of the cells in the absence of the staining of the membrane of the non-transfected cells. The area of the cell nucleus was only slightly stained (Figure 1(2A)).
- Antibodies against MuSK produced a smooth-to-very fine granular fluorescence of the cells in the absence of the staining of the membrane of the non-transfected cells. The area of the cell nucleus was only slightly stained (Figure 1(3B)). To evaluate the BIOCHIP mosaic intra-laboratory reproducibility, positive and negative control samples were tested 10 times in separate runs by 2 blinded operators. The overall agreement within the intra-laboratory runs was close to 100%.

The Anti-AChR autoantibodies were positive (≥0.50 nmol/L) in 44 out of 71 (62%) by ELISA and in 27 out of 71 (38%) by the BIOCHIP mosaic (combining the results of fetal and adult AChR assays).

**Figure 2:**
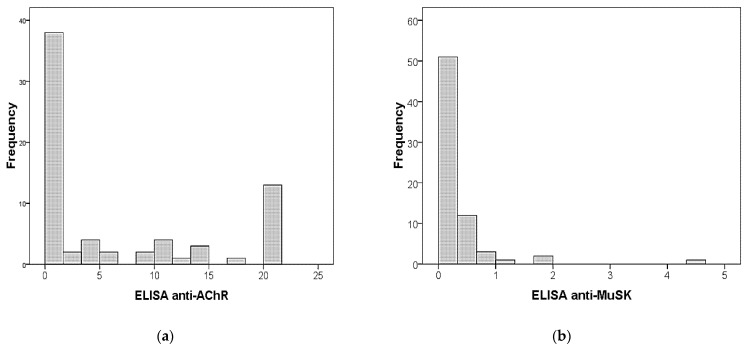
(**a**) Histogram of anti-AChR antibodies. Levels higher than 20 were represented here as having levels equal to 20; (**b**) histogram of anti-MuSK antibodies.

The median (IQR) levels of anti-AChR autoantibodies in ELISA were 1.25 (0.25–12.88), with 13 subjects displaying levels higher than 20 (Figure 2). Of note, 15 out of 17 patients, whose diagnoses of MG were not identified by the BIOCHIP mosaic, with a lack of AChR positivity, had a low autoantibodies titer on the ELISA method, whilst the other 2 patients had a higher median anti-AChR titer.

Anti-MuSK antibodies were considered as positive (≥0.50 nmol/L) in 11 out of 71 (15%) by ELISA and in 2 out of 71 (4%) by the BIOCHIP mosaic. Similarly, anti-MuSK negative patients on the BIOCHIP mosaic had a low antibodies titer, whilst anti-MuSK positive patients on the BIOCHIP mosaic had higher antibodies levels (Figure 2).

Patients with anti-AChR and anti-MuSK double positivity in the ELISA and BIOCHIP mosaic were respectively 5 (7%) and 0 (0%). A total of 20 MG patients (28.2%) were seronegative tested by ELISA, while 41 (57.7%) tested by BIOCHIP. We also associated either the ELISA positivity or the fluorescence patterns observed by BIOCHIP with the clinical manifestations (OMG or GMG) of each patient. This clinical information was available only in 58 (41 GMG and 17 OMG) patients. Positivity to anti-AChR autoantibodies by ELISA was found in 26/41 (63%) patients with GMG and in 8/17 (47%) patients with OMG; no significant association was evident (Fisher’s exact test *p* = 0.380). The AChRE positive pattern was found in 18/41 (44%) patients with GMG and in 2/17 (12%) patients with OMG. Similarly, the AChRG positive pattern was present in 20/41 (49%) of GMG patients and in only 2/17 (12%) with OMG. A statistically significant association was found between the clinical manifestation and both the AchRE or the AchRG pattern (Fisher’s exact test, respectively, *p* = 0.032 and *p* = 0.009). Anti-MuSK positivity by ELISA was present in 5/41 (12%) patients with GMG and in 3/17 (18%) patients with OMG; no significant association was found (Fisher’s exact test *p* = 0.681). A MuSK positive pattern was observed in only 2/41 (5%) of GMG patients.

The cross-tabulation of anti-AChR positivity in ELISA and IIF (fetal and/or adult AChR) is shown in Table 1. The overall concordance between ELISA and IIF for anti-AChR reactivity, calculated as concordant pairs, was (26 + 26)/70 = 74%. Cohen’s kappa was 0.51 (95% CI 0.32–0.71), which corresponds to 90% of the maximum possible kappa (0.57), given the observed marginal frequencies.

The cross-tabulation of anti-MuSK positivity in ELISA and IIF is shown in Table 2. The overall concordance between ELISA and IFI for anti-MuSK reactivity, calculated as concordant pairs, was (58 + 1)/70 = 84%. Cohen’s kappa was 0.11 (95% CI 0.00–0.36), which corresponds to 41% of the maximum possible kappa (0.27), given the observed marginal frequencies.

## 4. Discussion

**Figure 3:**
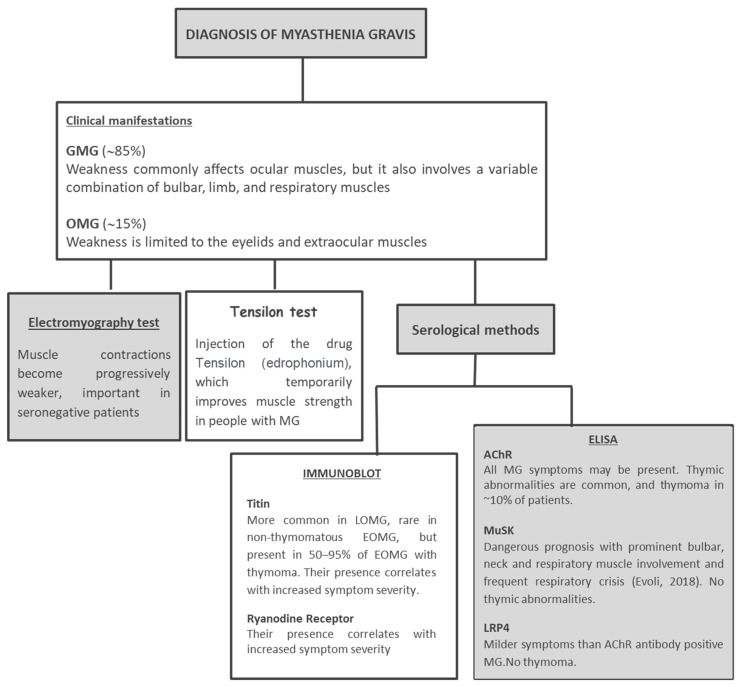
The algorithm for the diagnosis of MG. Serological antibody testing is the gold standard for the diagnosis of MG. Autoantibodies identification is important for designing better diagnosis.

The diagnosis of MG is based on a multistep approach, which combines clinical features and laboratory criteria (Figure 3). The sensitive and specific detection of anti-AChR and anti-MuSK autoantibodies has become an essential laboratory investigation in evaluating patients with MG, because seropositivity has diagnostic, prognostic, and therapeutic implications [15,16,17,18,19].

Recent studies report that anti-MuSK positive patients have a worse prognosis with predominant bulbar and respiratory muscle involvement and frequent respiratory crises [3,20,21,22]. Moreover, they respond poorly to acetylcholinesterase inhibitors, and conventional pyridostigmine doses frequently induce side effects, while they respond well to corticosteroids and to many steroid-sparing agents [23,24]. In addition, Rituximab should be considered as an early therapeutic option in patients who have an unsatisfactory response to initial immunotherapy [25,26]. Anti-MuSK autoantibodies primarily belong to the IgG4 subclass, which does not actives complement but rather directly inhibits protein function [27,28]. Interestingly, their titers positively correlate with symptom severity and a decrease in their response to plasma exchange [29]. Unlike anti-MuSK antibodies, anti-AChR autoantibodies primarily belong to the IgG1 and IgG3 subclasses and active complement cascades, causing the destruction of the post-synaptic membrane [30,31]. Their titers, generally, do not correlate with disease severity; however, the temporal variation of anti-AChR autoantibodies levels in a patient can be associated with symptom severity and the response to treatment as well. Thus, anti-AChR and anti-MuSK antibodies are associated to distinct disease entities with unique immunopathologic mechanisms.

Over the past decades, several analytical methods have been developed to measure anti-AChR and anti-MuSK autoantibodies, such as RIPA and ELISA [32,33,34]. However, these methods have some important analytical drawbacks, such as the use of radioactive isotopes for RIPA or a long TAT, the lack of standardization, and excessive cost for ELISA, resulting in an inconvenience for small laboratories. Different types of ELISA have been developed based either on direct or competitive methods, as shown in Table 3. Recently, a new method, the BIOCHIP mosaic, which simultaneously detects anti-AChR and anti-MuSK autoantibodies, has been developed. Although the BIOCHIP mosaic does not provide quantitative autoantibody serum levels, the simultaneous and multi-parametric analysis of all relevant antibodies provides faster results and a more cost-effective approach. Moreover, the BIOCHIP mosaic allows the discrimination between antibodies directed against fetal or adult AChR, which is important for the diagnosis of transient neonatal MG [35]. This condition is characterized by the presence in the mother of antibodies against the fetal AChR form only, which could not cause symptoms of MG in the mother, but could be harmful for the newborn [36,37].

In this study, for the first time, we compared the performance of a new multiparametric BIOCHIP mosaic-based IIF technique with ELISA for anti-AChR and anti-MuSK autoantibodies evaluation. Our results showed a concordance between ELISA and the BIOCHIP mosaic of 74% and 84% for anti-AChR reactivity and anti-MuSK reactivity, respectively. The Cohen’s kappa was 0.51 (95% CI 0.32–0.71) and 0.11 (95% CI 0.00–0.36) for anti-AChR and anti-MuSK reactivity, respectively. Thus, the overall concordance between ELISA and the BIOCHIP mosaic is not optimal. Interestingly, we observed a significant association between the clinical manifestation and both the AChRE or the AChRG pattern, suggesting a possible discriminatory power between ocular and generalized MG by using the BIOCHIP method.

A number of studies assessed the validity of the BIOCHIP mosaic method for detecting anti-desmoglein 1 and 3 antibodies in the diagnosis of autoimmune bullous diseases [38,39,40,41,42,43], presenting encouraging results. In addition, only one study reported the BIOCHIP mosaic assay as a powerful tool for the diagnosis of neuromyelitis optica [44].

Unfortunately, our findings showed that the BIOCHIP mosaic for MG diagnosis did not accurately identify specific autoantibodies. In particular, we observed that low autoantibody titrrs, detected by ELISA, were undetectable by the BIOCHIP method and, thus, the patients were identified as “seronegative”.

In conclusion, although BIOCHIP has great potential, since it offers different advantages, being easy to perform and less-time consuming, eliminating the need for multiple runs, more efforts are required to improve the antibodies detection in order to use it as an alternative test to ELISA and RIPA in MG diagnosis.

## Figures and Tables

**Table 1:** The analysis of the agreement between BIOCHIP and ELISA assays for the detection of anti-AChR antibodies.

	ELISA Anti-AChR			
BIOCHIP IIF	Positive	Negative	Total	BIOCHIP IIF
Positive	26	1	27	Positive
Negative	17	26	43	Negative
Total	43	27	70	Total

**Table 2:** The analysis of the agreement between BIOCHIP IIF and ELISA assays for the detection of anti-MuSK antibodies.

	ELISA anti-MuSK			
BIOCHIP IIF	Positive	Negative	Total	Kappa
Positive	1	1	2	
Negative	10	58	68	0.11
Total	11	59	70	

**Table 3:** Summary of commercially ELISA assays for anti-AChR detection.

Name	Assay Type	Sensitivity	Detection Range	Company	Regulatory Status
Human AChR-Ab	Quantitative Sandwich	0.04 pmol/mL	0.06–4 pmol/mL	MyBioSource	RUO
Anti-AChR Ab	Competitive	0.23 nmol/L	0.2–20 IU/mL	Eagle Bioscience	RUO
Human Anti-AChR Ab	Indirect	0.938 ng/mL	1.563–100 ng/mL	Biomatik	RUO
Medizym^®^ Anti-AChR Ab	Competitive	0.23 U/mL	0.2–20 U/mL	Medipan	IVD
Anti-AChR Ab	Competitive	0.25 nmol/L	0.2–20 nmol/L	RSR Limited	IVD
Anti-AChR Ab	Indirect	0.11 nmol/L	0–8 nmol/L	Euroimmun	IVD

AChR-Ab, Acetylcholine Receptor Antibody; AChR, Acetylcholine Receptor; RUO, Research Use Only; and IVD, In Vitro Diagnostic.

## Data Availability

Derived data supporting the findings of this study are available from the corresponding author on request.
